# Is a positive Christie-Atkinson-Munch-Peterson (CAMP) test sensitive enough for the identification of *Streptococcus agalactiae*?

**DOI:** 10.1186/s12879-018-3561-3

**Published:** 2019-01-03

**Authors:** Dacheng Guo, Yu Xi, Shanmei Wang, Zeyu Wang

**Affiliations:** 1 Institute for Infectious Disease Control and Prevention, Henan Provincial Center for Disease Control and Prevention, Zhengzhou, China; 2 School of Life Sciences, Zhengzhou University, Zhengzhou, China; 3 Department of Clinical Laboratory, Henan Provincial People’s Hospital, Zhengzhou, China; 4 R&D Center, Autobio Diagnostics Co. Ltd, Zhengzhou, China

**Keywords:** CAMP test, *Streptococcus agalactiae*, PCR, 16S rDNA gene sequencing

## Abstract

**Background:**

For a long time, the Christie-Atkinson-Munch-Peterson (CAMP) test has been a standard test for the identification of *Streptococcus agalactiae*, and a positive result for *S.agalactiae* has been considered sensitive enough.

**Methods:**

To confirm whether a positive CAMP test is a requirement for the identification of *S.agalactiae*, five suspected CAMP-negative *S.agalactiae* isolates from two hospitals, confirmed as Gram-positive and catalase-negative streptococci, were verified by the CAMP test in three batches of plates from two manufacturers and identified by the Phoenix system, MALDI-TOF MS, the PCR assay and the 16S rDNA gene sequencing.

**Results:**

All five suspected strains were *S.agalactiae*, four of which were CAMP-negative and one of which was not *S.agalactiae* by the PCR assay.

**Conclusions:**

A positive CAMP test was lacking sensitivity for the identification of *S.agalactiae*, and the question of whether the *cfb* gene is worthy of targeting should be further studied.

## Background

*Streptococcus agalactiae*, also known as Group B Streptococcus (GBS), causing intrapartum and postpartum infection, is the most common cause of neonatal sepsis, and has a high mortality rate [1–4]. In view of the dangers of GBS, prenatal GBS screening of pregnant women at 35 to 37 weeks’ gestation has been recommended by the competent departments of health in many countries [5–8]. The CAMP (Christie, Atkinson, Munch, Peterson) test is used in some laboratories to verify whether bacteria have enhanced staphylococcus beta-lysis activity test, which has long been considered as a key, confirmed test for the identification of GBS [9–12]. Some PCR-based assays for GBS have targeted the *cfb* gene, which encodes the CAMP factor [13, 14].

However, a positive CAMP test for GBS seemed to be lacking sensitivity. From the beginning of the twenty-first century, several studies have isolated the CAMP-negative GBS [15, 16], but it did not attract much attention from scholars in clinical medicine. Those academic achievements were generally derived from the same team; furthermore, all of those studies were performed on bovine mastitis. Until 2016, CAMP-negative GBS was only reported to be found in clinical laboratories [17]. In this paper we present evidence CAMP-negative GBS did exist, and not just in low proportions.

## Methods

### GBS isolates

Four isolates of suspected GBS and CAMP-negative strains, sequentially numbered 50, 51, 53 and 54, were obtained from the vaginal swabs of 174 patients admitted to the Shenzhen Maternity & Child Healthcare Hospital by using the GBS chromogenic agar plates and the blood agar plates (BAPs) (Autobio Diagnostics, Zhengzhou, China) in October 2016, where 22 samples of suspected GBS were isolated. Another isolate of suspected GBS and CAMP-negative origin, numbered 49, was obtained from 286 patients admitted to the Zhengzhou Maternity & Child Healthcare Hospital in November 2016, where 27 samples of suspected GBS were isolated. All five suspected strains were identified as Gram-positive and catalase-negative streptococci.

### CAMP test

BAPs used in the study were derived from two manufacturers in three batches. Batch numbers 20161228B-Y, 20161104B and 20161117B came from Autobio Diagnostics (Zhengzhou, China), and batch numbers XWO1601811, XWO1601812 and XWO1601838 came from OXOID (Beijing, China). *Staphylococcus aureus* (ATCC25923) was streaked in the middle of the BAPs, and the lines of each of the suspected strains, as well as the QC strains, were streaked perpendicularly to *S.aureus*. GBS (ATCC13813) and *Streptococcus pyogenes* (ATCC19615) were used as positive and negative QC, respectively. The distance between two lines perpendicular to each other was approximately 1–2 mm. The plates were incubated aerobically at 35–37 °C for 18–24 h after inoculation. An arrow-head shaped zone of beta-haemolysis at the junction of perpendicular lines constituted a positive CAMP reaction.

### Bacterial identification by four assays

To confirm whether the five strains were GBS, bacterial identification was carried out by the following four assays: the biochemical identification system, the matrix-assisted laser desorption ionisation time-of-flight mass spectrometry (MALDI-TOF MS), the PCR assay and the 16S rDNA gene sequencing.

The Phoenix™—100 ID/AST system (Becton Dickinson, Sparks, USA) was adopted as a biochemical identification system, and isolates activated on BAPs (Autobio Diagnostics, Zhengzhou, China) were inoculated in a 0.5 McFarland suspension into the SMIC/ID-2 cards.

MALDI-TOF MS analysis of activated isolates was performed on a Bruker Microflex (Bruker Daltonics, Bremen, Germany) using the Flexcontrol software (version 3.4). A colony was directly spotted on a 96-spot polished steel target plate in duplicates, and was pipetted in 1 μl of matrix solution (ultra pure water: 475 μl, formic acid: 25 μl, acetonitrile: 500 μl, α-cyano-4-hydroxycinnamic acid: 10 mg) was added after drying. Then, the target plate was dried again and loaded on the instrument, measured and analysed. An *Escherichia coli* DH5-α standard (Bruker Daltonics, Bremen, Germany) was used for external calibration, and the identification results were presented in the MALDI biotyper 3.0 as the log score values.

The PCR assay was performed using the Group B Streptococcus Nucleic Acid Detection Kit (Fluorescent PCR, Lot: 241611, Triplex, Fuzhou, China) that targets the *cfb* gene, in strict accordance with the manufacturer’s instruction. The concentration of activated isolates used for analysis was 10^4^ CFU/ml.

The genomic DNA was isolated and purified from cells after activation by using SK 8255 (Sangon Biotech, Shanghai, China), a genomic extraction kit, as per the manufacturer’s instructions. DNA 16S region amplification was performed using the primer set 7F-1540R. The 16S rDNA gene (~ 1500 bp) was amplified employing universal primers (7F 5’-CAGAGTTTGATCCTGGCT-3′, 1540R 5’-AGGAGGTGATCCAGCCGCA-3′). Amplification was carried out on the Applied Biosystems 2720 thermal cycler (ThermoFisher, Foster City, USA) according to the manufacturer’s recommendations. The amplified fragment was purified from the agarose gel with SK8131 (Sangon Biotech, Shanghai, China), the gel extraction kit, as per the manufacturer’s instructions. The amplified product was sequenced by a standard procedure on the Applied Biosystems 3730 sequencer (ThermoFisher, Foster City, USA). The obtained 16S rDNA sequence data were aligned and compared with similar sequences from the GenBank database of NCBI using the BLAST program.

GBS (ATCC13813) was used as positive QC, *S.pyogenes* (ATCC19615) and *S.aureus* (ATCC25923) were included as negative QC as well in these four assays.

## Results

**Fig. 1:**
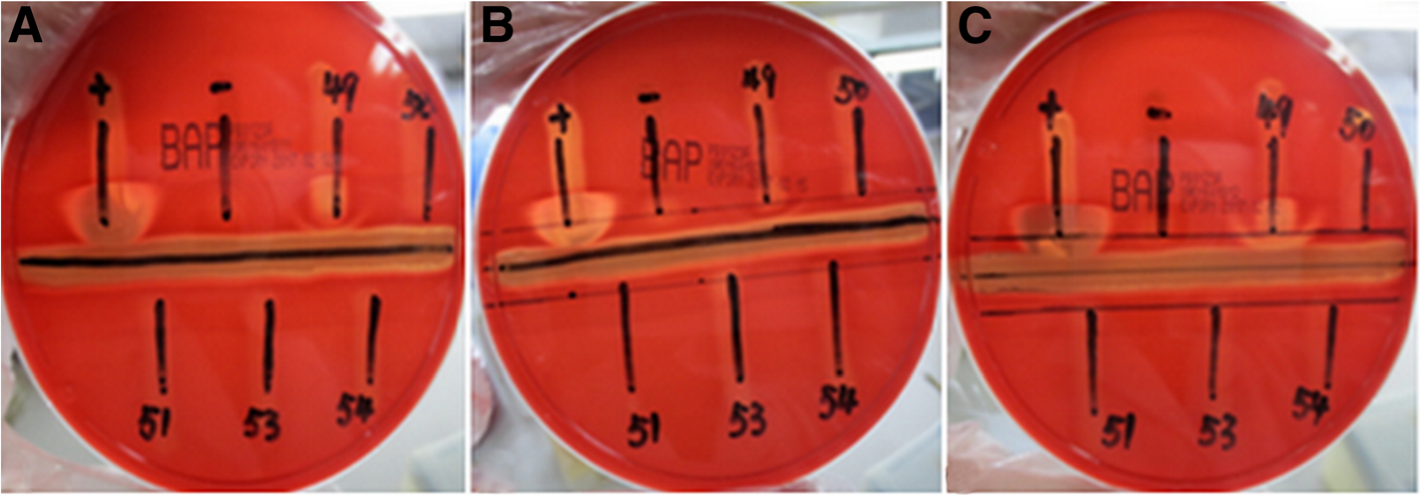
The CAMP test of five suspected strains on three batches of BPAs from OXOID (A: XWO1601811, only No.49 was weakly positive, B: XWO1601838, only No.49 was almost no positive, C: XWO1601812, only No.49 was positive)

**Fig. 2:**
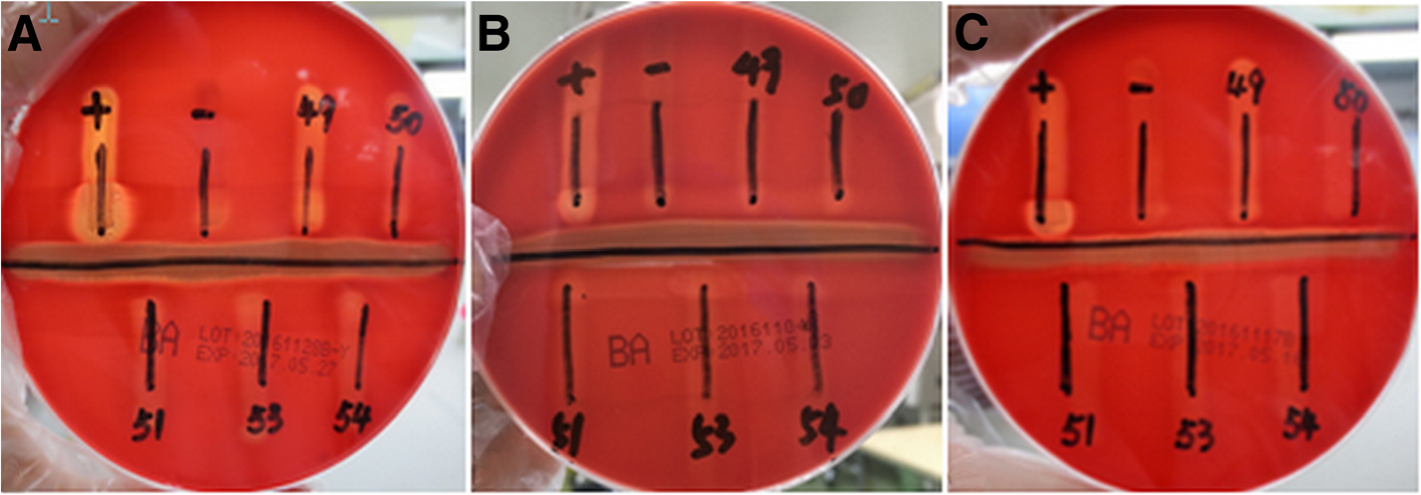
The CAMP test of five suspected strains on three batches of BPAs from Autobio (A: 20161228B-Y, B: 20161104B, C: 20161117B, only No.49 was positive on BPAs of batch number 20161228B-Y)

Four suspected strains numbered 50, 51, 53 and 54, were clearly demonstrated to be CAMP-negative on six different batches of BAPs from two different manufacturers, but the results of strain No.49 differed (Fig. 1 and Fig. 2). This isolate was CAMP-negative on two batches of BAPs from Autobio and CAMP-positive on the third batch (20161128B-Y), while all three batches of OXOID, demonstrated an extremely weak or obvious arrow-head shape of the zone of enhanced haemolytic activity.

Strain No.51 could not be identified as GBS by the PCR assay only, however, all other four suspected strains were GBS by all four methods. Strain No.51 was identified as GBS by all the other three methods (Table 1). Compared with six similar 16S rDNA sequences (Accessions numbers: CP016501, CP010875, CP010874, CP010319, CP011327 and CP011326) from GenBank, the sequence of No.51 had the ident of 99% with all six sequences. However, all the other four strains had the ident of 100%.

**Table 1:** ID results of five suspected strains by four methods

Strain numbers	Biochemical identification		Fluorescent PCR		MALDI-TOF MS		16S rDNA identification	
	Confidence (%)	ID results	Ct values^*^	ID results	Log score values	ID results	Identity (%)	ID results
49	99	GBS	23 (positive)	GBS	2.221	GBS	100(6)^**^	GBS
50	98	GBS	28 (grey zone)	GBS	2.478	GBS	100(6)	GBS
51	98	GBS	No Ct	No GBS	2.317	GBS	99(6)	GBS
53	99	GBS	27 (grey zone)	GBS	2.236	GBS	100(6)	GBS
54	99	GBS	27 (grey zone)	GBS	2.169	GBS	100(6)	GBS
ATCC 13813	99	GBS	23 (positive)	GBS	2.436	GBS	100(5) & 99(1)	GBS
ATCC 19615	99	*S.pyogenes*	No Ct	No GBS	2.382	*S.pyogenes*	100(6)	*S.pyogenes*
ATCC 25923	0	UNIORG***	No Ct	No GBS	2.357	*S.aureus*	100(6)	*S.aureus*

*Positive control: < 23, Negative control: =30 or No Ct

**Figures in brackets denote the number of similar sequences

***SMIC/ID-2 cards are dedicated to the identification of streptococcus and cannot identify staphylococci

## Discussion

Many diagnostic guidelines still recommend using a positive CAMP test as a significant condition for identification of GBS, so the resulting problem of failing to detect some GBS is given insufficient attention. The result of the CAMP test may be affected by many factors, such as culture conditions, culture time, and culture temperature [18, 19], but once these conditions are standardised, the most likely factor is the quality of the BPAs. Ensuring strict quality control and using multiple batches of products from different manufacturers to eliminate the effects of medium and inoculation, the detection rate of 18.18% (4/22) in Shenzhen suggest that CAMP-negative GBS not only existed in some parts of China but also were detected in large numbers. This proportion was higher than that reported in the literature [20]. To confirm these findings, additional studies with expanded sample sizes are needed.

The PCR kit used in this study targeted the *cfb* gene. To eliminate the false negatives due to insufficient sensitivity of the kit, we increased the concentration of bacterial suspensions to four McFarland; however, the sample No.51 was still negative in these conditions. These results suggest that at least some CAMP-negative GBS may not carry the *cfb* gene, which is different from the traditional view that the *cfb* gene is present in every GBS isolate [21, 22]. Strains with the *cfb* gene may also produce the CAMP-negative phenotype because the *cfb* gene is transcriptionally defective [23], gene expression is low, or the activity of the CAMP factor expression is low. The CAMP factor of GBS is a virulent protein with a complete sequence of 226 amino acids [24], but it is not essential for the systemic virulence of GBS [25], and some studies propose the existence of a different CAMP factor, named CAMP factor II [26].

In recent years, the culture-free GBS detection techniques have developed rapidly [27–30]. Since the CAMP factor can bind in a non-immune reaction to the IgG and IgM antibody classes in various mammalian species [31], it is not a suitable antigen for immunoassays. However, the *cfb* gene is the most commonly used target for PCR assays. Our research suggest that targeting the *cfb* gene or the CAMP factor results in missing CAMP-negative GBS. Since CAMP-negative GBS do exist, the question of whether the *cfb* gene is worthy of targeting should be further studied, especially if the number of these GBS is relatively high.

## Conclusions

A positive CAMP test was lacking sensitivity for the identification of GBS, and the question of whether the *cfb* gene is worthy of targeting should be further studied.

## Abbreviations

BAPs: blood agar plates
CAMP: Christie-Atkinson-Munch-Peterson
GBS: Group B Streptococcus
MALDI-TOF MS: matrix-assisted laser desorption ionisation time-of-flight mass spectrometry
